# QuickStats

**Published:** 2015-04-03

**Authors:** FROM THE NATIONAL CENTER FOR HEALTH STATISTICS

**Figure:**
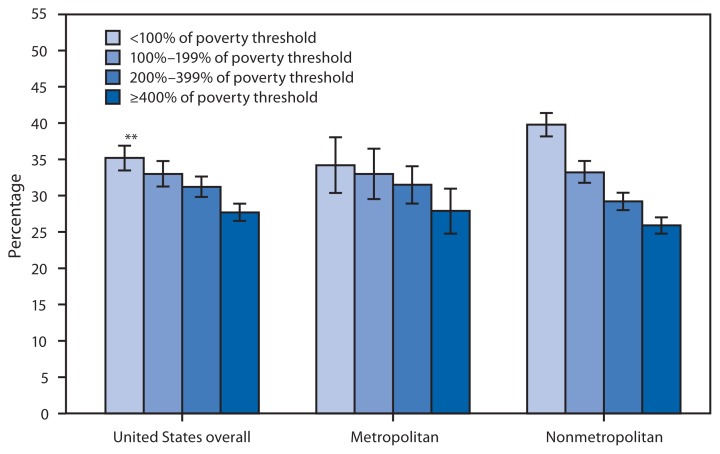
Percentage of Adults Who Average ≤6 Hours of Sleep,* by Family Income Group^†^ and Metropolitan Status of Residence^§^ — National Health Interview Survey, United States, 2013¶ ^*^ Participants were asked, “On average, how many hours of sleep do you get in a 24-hour period?” ^†^ Family income groups were defined based on family income as a percentage of the federal poverty threshold. Poverty thresholds, which are published by the U.S. Census Bureau, vary by family size and the number of children in the family. Family income was imputed when missing using multiple imputation methodology. ^§^ Based on the household residence location. Metropolitan is located within a metropolitan statistical area, defined as a county or group of contiguous counties that contains at least one urbanized area of ≥50,000 population. Surrounding counties with strong economic ties to the urbanized area also are included. Nonmetropolitan areas do not include a large urbanized area and are generally thought of as more rural. ^¶^ Estimates are based on household interviews of a sample of the civilian, noninstitutionalized U.S. population and are derived from the National Health Interview Survey sample adult component. ^**^ 95% confidence interval.

During 2013, the percentage of adults who slept ≤6 hours in an average 24-hour period declined with family income from 35.2% for those with family incomes <100% of the poverty level to 27.7% for those with family incomes ≥400% of the poverty level. The same pattern was found for those living in metropolitan and nonmetropolitan areas. There were no statistically significant differences between those living in metropolitan and nonmetropolitan areas except among those with family incomes <100% of the poverty level, where 39.8% of adults living in nonmetropolitan areas slept ≤6 hours compared with 34.2% of adults living in metropolitan areas.

**Source:** National Health Interview Survey, 2013 data. Available at http://www.cdc.gov/nchs/nhis.htm.

**Reported by:** Lindsey I. Black, MPH, LBlack1@cdc.gov, 301-458-4548; Renee M. Gindi, PhD.

